# Practical Guidelines for the Comprehensive Analysis of ChIP-seq Data

**DOI:** 10.1371/journal.pcbi.1003326

**Published:** 2013-11-14

**Authors:** Timothy Bailey, Pawel Krajewski, Istvan Ladunga, Celine Lefebvre, Qunhua Li, Tao Liu, Pedro Madrigal, Cenny Taslim, Jie Zhang

**Affiliations:** 1 Institute for Molecular Bioscience, The University of Queensland, Brisbane, Australia; 2 Department of Biometry and Bioinformatics, Institute of Plant Genetics, Polish Academy of Sciences, Poznań, Poland; 3 Department of Statistics, Beadle Center, University of Nebraska-Lincoln, Lincoln, Nebraska, United States of America; 4 Inserm U981, Cancer Institute Gustave Roussy, Villejuif, France; 5 Department of Statistics, Penn State University, University Park, Pennsylvania, United States of America; 6 Department of Biochemistry, University at Buffalo, Buffalo, New York, United States of America; 7 Department of Biomedical Informatics, The Ohio State University, Columbus, Ohio, United States of America; Whitehead Institute, United States of America

## Abstract

Mapping the chromosomal locations of transcription factors, nucleosomes, histone modifications, chromatin remodeling enzymes, chaperones, and polymerases is one of the key tasks of modern biology, as evidenced by the Encyclopedia of DNA Elements (ENCODE) Project. To this end, chromatin immunoprecipitation followed by high-throughput sequencing (ChIP-seq) is the standard methodology. Mapping such protein-DNA interactions *in vivo* using ChIP-seq presents multiple challenges not only in sample preparation and sequencing but also for computational analysis. Here, we present step-by-step guidelines for the computational analysis of ChIP-seq data. We address all the major steps in the analysis of ChIP-seq data: sequencing depth selection, quality checking, mapping, data normalization, assessment of reproducibility, peak calling, differential binding analysis, controlling the false discovery rate, peak annotation, visualization, and motif analysis. At each step in our guidelines we discuss some of the software tools most frequently used. We also highlight the challenges and problems associated with each step in ChIP-seq data analysis. We present a concise workflow for the analysis of ChIP-seq data in **Figure 1** that complements and expands on the recommendations of the ENCODE and modENCODE projects. Each step in the workflow is described in detail in the following sections.

## Introduction to ChIP-seq Technology

Chromatin immunoprecipitation followed by sequencing (ChIP-seq), first described in 2007 [1]–[4], allows *in vivo* determination of where a protein binds the genome, which can be transcription factors, DNA-binding enzymes, histones, chaperones, or nucleosomes. ChIP-seq first cross-links bound proteins to chromatin, fragments the chromatin, captures the DNA fragments bound to one protein using an antibody specific to it, and sequences the ends of the captured fragments using next-generation sequencing (NGS). Computational mapping of the sequenced DNA identifies the genomic locations of bound DNA-binding enzymes, modified histones, chaperones, nucleosomes, and transcription factors (TFs), thereby illuminating the role of these protein-DNA interactions in gene expression and other cellular processes. The use of NGS provides relatively high resolution, low noise, and high genomic coverage compared with ChIP-chip assays (ChIP followed by microarray hybridization). ChIP-seq is now the most widely used procedure for genome-wide assays of protein-DNA interaction [5], and its use in mapping histone modifications has been seminal in epigenetics research [6].[Fig pcbi-1003326-g001]

**Figure 1 pcbi-1003326-g001:**
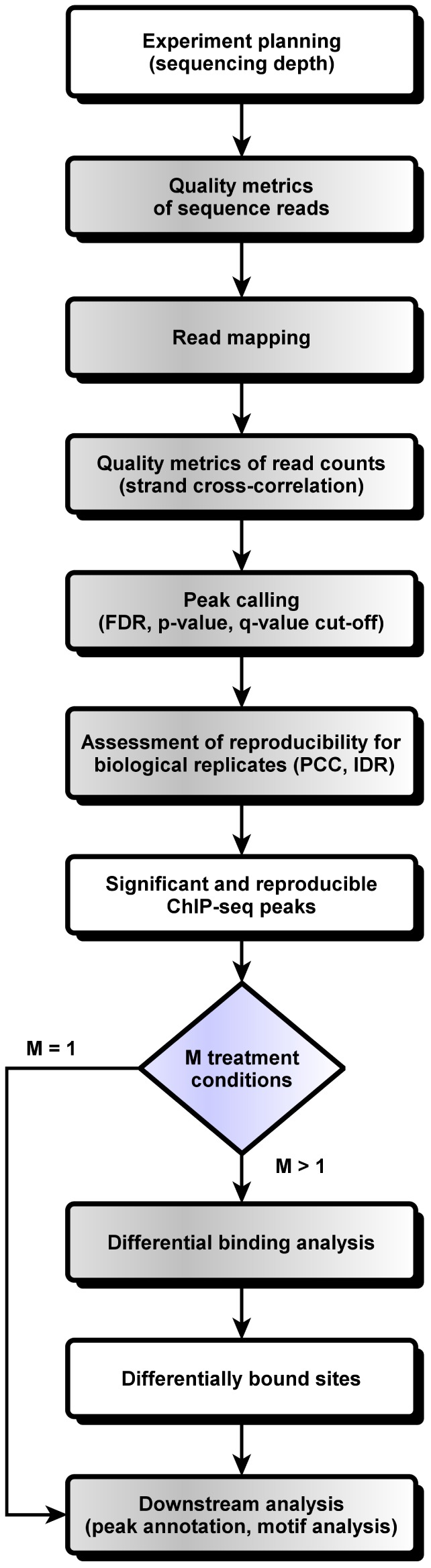
Workflow for the computational analysis of ChIP-seq.

## The Analysis of ChIP-seq Data

### Sequencing Depth

Effective analysis of ChIP-seq data requires sufficient coverage by sequence reads (sequencing depth). The required depth depends mainly on the size of the genome and the number and size of the binding sites of the protein. For mammalian transcription factors (TFs) and chromatin modifications such as enhancer-associated histone marks, which are typically localized at specific, narrow sites and have on the order of thousands of binding sites, 20 million reads may be adequate (4 million reads for worm and fly TFs) [7]. Proteins with more binding sites (e.g., RNA Pol II) or broader factors, including most histone marks, will require more reads, up to 60 million for mammalian ChIP-seq [8]. Importantly, control samples should be sequenced significantly deeper than the ChIP ones in a TF experiment and in experiments involving diffused broad-domain chromatin data. This is to ensure sufficient coverage of a substantial portion of the genome and non-repetitive autosomal DNA regions. To ensure that the chosen sequencing depth was adequate, a saturation analysis is recommended—the peaks called should be consistent when the next two steps (read mapping and peak calling) are performed on increasing numbers of reads chosen at random from the actual reads. Saturation analysis is built into some peak callers (e.g., SPP [9]). If this shows that the number of reads is not adequate, reads from technical replicate experiments can be combined. To avoid over-sequencing and estimate an optimal sequencing depth, it is important to take into account library complexity. Several tools are available for this purpose. For example, the preseq package allows users to predict the number of redundant reads from a given sequencing depth and how many will be expected from additional sequencing [10]. Similarly, the ENCODE software tools offer a quality metric called the PCR bottleneck coefficient (PBC), defined as the fraction of genomic locations with exactly one unique read versus those covered by at least one unique read.

### Read Mapping and Quality Metrics

Before mapping the reads to the reference genome, they should be filtered by applying a quality cutoff (**Box 1**). The remaining reads should then be mapped using one of the available mappers such as Bowtie [11], BWA [12], SOAP [13], or MAQ [14]. Recent versions support gapped alignment (e.g., Bowtie2), but detection of indels is not necessary for most ChIP-seq experiments. It is important to consider the percentage of uniquely mapped reads reported by the mapper. The percentage varies between organisms, and for human, mouse, or *Arabidopsis* ChIP-seq data, above 70% uniquely mapped reads is normal, whereas less than 50% may be cause for concern. A low percentage of uniquely mapped reads often is due either to excessive amplification in the PCR step, inadequate read length, or problems with the sequencing platform, but with some ChIPed proteins it may be unavoidable (e.g., if the protein binds frequently in repetitive DNA). The read mappers are designed to allow a (user-settable) number of mismatches in the reads, and it is important to choose this parameter to be appropriate with the NGS platform being used (consult the manufacturer). A final potential cause of high numbers of “multi-mapping” reads is that the protein binds frequently in regions of repeated DNA. In this last case, using paired-end sequencing to reduce the mapping ambiguity may help. It should be kept in mind that multi-mapping reads will be ignored (filtered out) by most peak-calling algorithms (see section “Peak Calling”), although they can drive the discovery of novel binding sites [15].

**Box 1. Quality metrics of sequence reads**

Preprocessing of ChIP-seq data will, in general, be similar to that of any other sequencing data and will assess the quality of the raw reads to identify possible sequencing errors or biases (FastQC can be used for an overview of the data quality). Phred quality scores are used to describe the confidence of each base call in each sequence tag, are logarithmically linked to error probabilities, and can be used to filter low-quality reads. After this filtering step, it may also be necessary to trim the end of reads that are of low quality (see sickle, https://github.com/najoshi/sickle). Additionally, library complexity is a common quality measure for ChIP-seq libraries (preseq package [10] or PCR bottleneck coefficient [PBC] from ENCODE tools, https://code.google.com/p/phantompeakqualtools/), and library complexity is linked to many factors such as antibody quality, over-cross-linking, amount of material, sonication, or over-amplification by PCR. The latter can be corrected by systematic identification and removal of redundant reads, which is implemented in many peak callers as it may improve their specificity. Readers may be interested in the Galaxy toolbox, which offers access to many of the tools described here [50].

After mapping, the signal-to-noise ratio (SNR) of the ChIP-seq experiment should be assessed, for example via quality metrics such as strand cross-correlation [7] or IP enrichment estimation using the software package CHANCE [16] (**Box 2**). These measures will detect several possible failure modes of ChIP-seq: insufficient enrichment by immunoprecipitation step, poor fragment-size selection, or insufficient sequencing depth. Strand cross-correlation analysis is built into some peak callers (e.g., SPP or MACS [17] [version 2]).

**Box 2. Quality metrics of read counts**

Strand cross-correlation analysis [7] assesses data quality by measuring the degree of immunoprecipitated (IP) fragment clustering in ChIP-seq experiments. It is developed based on the observations that (1) a high-quality ChIP-seq experiment often shows a significant clustering of enriched DNA sequence tags at the locations bound by the protein of interest, and that (2) the enriched sequence tags on the forward and reversed strands are positioned at a distance from the binding site center that depends on the fragment size distribution [9]. This method quantifies the degree of clustering by computing the cross-correlation between the two strands, i.e., the Pearson correlation between the strand-specific read density profiles as a function of the shift (*k*) applied to one of the two strands (**Figure S1**). The cross-correlation typically peaks at the shift corresponding to the fragment length and the shift corresponding to the read length. The ratio between the cross-correlation at the fragment length and the background cross-correlation, referred to as normalized strand cross-correlation coefficient (NSC), and the ratio between cross-correlation at the fragment length and the cross-correlation at the read length, referred to as relative strand cross-correlation coefficient (RSC), jointly reflect signal-to-noise ratio in the ChIP-seq data. Very successful ChIP experiments generally have NSC>1.05 and RSC>0.8 [7], although there can still be significant biological information present in ChIP-seq data not meeting these criteria. Readers may refer to [7] for prototypical profiles of cross-correlation illustrated on ENCODE data.

The software CHANCE [16] assesses IP strength by estimating and comparing the IP reads pulled down by the antibody and the background, using a method called signal extraction scaling [77]. For each sample, it first bins the genome into non-overlap bins both for the IP and the Input, then partitions the bins into a signal region and a background region by comparing the cumulative distributions of tag counts in the bins of the IP and the Input. It next computes a *p*-value for significance of enrichment according to the percentage allocation of reads in each type of regions. Based on the empirical *p*-value distribution computed from a set of ENCODE IP-Input and Input-input experiments on human data, it estimates a *q*-value by treating the two types of experiments as true positives and false positives, respectively. The *q*-value thus is interpreted as the fraction of comparisons with ENCODE data that show differential enrichment at the level of the user's data but turn out to be technical replicates of the Inputs. The software determines the success of the experiment based on the *q*-values, and also reports some descriptive quality statistics, such as the percentage increase in mean tag density in IP compared to Input and the percentage of the genome classified as signal region. Because the *q*-values are computed based on human data, users should be aware that the *q*-values may not be relevant if their data are generated from other organisms.

CHANCE also provides a graphical visualization of IP strength with genome coverage, by plotting the empirical cumulative percentage of tags covered by the bins that are sorted in an increasing order of read density for both the IP and the Input. By examining and comparing the IP and Input curves, one may identify quality issues, such as insufficient sequencing depth, amplification bias, and weak IP enrichment.

### Peak Calling

A pivotal analysis for ChIP-seq is to predict the regions of the genome where the ChIPed protein is bound by finding regions with significant numbers of mapped reads (peaks). A fine balance between sensitivity and specificity depends on choosing an appropriate peak-calling algorithm and normalization method (**Boxes 3** **–** **6**, **Table S1**, and [18], [19]) based on the type of protein ChIPed: point-source factors such as most TFs (**Box 3**), broadly enriched factors such as histone marks (**Box 4**), and those with both characteristics such as RNA Pol II (**Box 5**) [20]. It is strongly recommended that mapped reads from a control sample be used (e.g., from input DNA), although some peak callers can use GC content or mappability as information necessary to assess the level of non-specific or background binding. Duplicate reads (same 5′ end) can be removed before peak calling to improve specificity (**Box 7**). Although some peak callers support both single and paired-end reads (e.g., MACS), others are specifically designed to improve sensitivity and specificity in paired-end sequencing (e.g., SIPeS [21]). Existing peak callers have many user-settable parameters that can greatly affect the number and quality of the peaks called. For instance, the enrichment metric for most peak callers, such as *p*-value or FDR, could be hugely affected by the statistical model used, the sequencing depth, or the actual number of binding sites in the genome. Thus, using the same *p*-value or FDR threshold does not ensure that the numbers of peaks called are comparable across libraries and different peak callers [22]. A better approach is to threshold the irreproducible discovery rate (IDR) [23], which, along with motif analysis, can also aid in choosing the best peak-calling algorithm and parameter settings (see sections “Assessment of Reproducibility” and “Motif Analysis”).

**Box 3. Peak calling: Punctate-source transcription factors**

Nowadays, since ChIP-seq data of point-source factors are the most abundant type, most peak callers are designed and fine-tuned for these factors. Existing peak callers differ from each other in terms of signal smoothing and background modeling. Because DNA around interaction sites is more easily sheared, the ends of ChIPed DNA fragments would form footprints on DNA whose size is more related to protein-DNA interaction than to size selection during library preparation. Those peak callers able to capture this experiment-specific information can greatly improve accuracy of prediction. For example, peak callers SPP [9] and MACS [17] (version 2) use cross-correlation to find the lag between reads mapped to the minus and the plus strand as the size of actual protein-DNA interacting regions. After smoothing, background models are then used to remove noise either directly from the control sample or from features of the genome sequence such as GC content or mappability (BEADS [84]). Peaks are finally called above a user-defined SNR level. Models used for the statistical assessment of enriched regions (peaks) range from Poisson (CSAR [85]), local Poisson (MACS), negative binomial (CisGenome [56]) to zero-inflated negative binomial (ZINBA [86]), or even extend to more sophisticated machine learning modeling techniques such as Hidden Markov Model (HPeak [87] and BayesPeak [88]).

Most peak-calling algorithms apply a window-based method to detect peaks, so nearby binding events may be erroneously merged. To improve the spatial resolution of binding event predictions, several peak callers use peak shape as a clue. PeakSplitter [89] can look for local maxima in a broader region containing several sub-peaks. GPS [67] builds a probabilistic model of the distribution of ChIP-seq reads at given peak candidate regions to deconvolve nearby homotypic events. The R packages polyaPeak and NarrowPeaks can analyze the shape of the peaks to re-rank and narrow down the final peak list, respectively. These approaches are highly recommended as a post-processing step after general peak calling for point-source factors.

**Box 4. Peak calling: Broad enriched regions from histone marks**

Due to the increasing interest in epigenetic regulation, epigenetic marks such as histone modifications, DNA methylation, and chromatin remodeling factors are being explored through ChIP-seq. Some of these marks are enriched strongly in narrow genomic regions (e.g., H3K4me3 at gene promoters), and the peak callers appropriate for point-source factors (discussed in **Box 3**) can be used. However, most histone marks tend to have more broadly spreading and weaker patterns (e.g., H3K27me3). Several peak callers are specifically designed for predicting broad regions from ChIP-seq data, including SICER [90], CCAT [91], ZINBA, and RSEG [92]. Other peak callers including SPP, MACS (version 2), and PeakRanger [93] can also be used with this type of ChIP-seq data by using their options to increase “bandwidth” or to relax the “peak cutoff.”

For broad marks, the pattern of enrichment should be described as “domains” instead of “peaks” because there are no clearly defined peak summits. An alternative representation of the pattern of mapped reads is hierarchical: combining multiple levels of enrichment. For example, MACS (version 2) and Scripture [94] (originally designed for RNA-seq) can make narrow calls for strong enrichment inside broader calls for weak enrichment associated with domain boundaries.

**Box 5. Peak calling: Mixed signals**

There are also some factors (such as RNA Pol II) that bind to DNA in regions with bigger variation. It is known that some RNA Pol II complexes are stalled while others are moving along with active transcription [95]. In the first case, data ideally should be treated as for a point-source factor, whereas in the second case, the data should be treated as for factors with broad marks. An ideal algorithm should accommodate both patterns, which means peak calling should be more general. Some tools have options for both narrow and broad peak calling, such as SPP, MACS, ZINBA, and PeakRanger. However, with careful parameter tweaking any algorithm suitable for broad peak detection would work for this type of data.

**Box 6. Normalization**

Whether comparing one ChIP sample against input DNA (sonicated DNA), “mock” ChIP (non-specific antibody, e.g., IgG) in peak calling, or comparing a ChIP sample against another in differential analysis, there are linear and non-linear normalization methods available to make the two samples “comparable” (**Table S2**). Although many methodologies focus on normalization to a control sample, none of them make the distinction on the type of control samples used. An intuitive and commonly used linear normalization technique is called sequencing depth normalization. In this method the number of reads is multiplied by a scale factor to make the total reads in different samples the same (see [9], [96] for details). A slight modification of the method is used in PeakSeq [24], where a scale factor is estimated in a region (∼10 Kb) using linear regression. Many other existing methods also use a normalization factor to linearly scale samples, focusing on normalization against control samples (see for example CisGenome [56], MACS [17], and USeq [97]). Another scaling normalization method known as RPKM (Reads per Kilobase of sequence range per Million mapped reads) proposed in [98] adjusts for biases due to the higher probability of reads falling into longer regions.

A non-linear normalization adjusts for biases with non-linear trend. In a method described in [28] the data is normalized with respect to mean and variance using locally weighted regression (LOESS). It is based on the assumption that the effect of biological condition change does not cause global binding alterations. This assumption can be applied, for example, when comparing samples with different stages of disease progression, or on samples before and after a certain treatment (see section “Differential Binding Analysis”). A modified version of this non-linear normalization is implemented as MAnorm [34], assuming that peaks common in the two conditions do not undergo global changes. The R package called POLYPHEMUS [37] has also been developed, implementing two normalization methods: (1) the non-linear method described in [28] and (2) a Quantile normalization that makes the distribution in different samples the same. Normalization issues are, at present, not fully exploited although they might have a substantial impact on the results [28], [37], [99].

**Box 7. Duplicated reads**

Duplicate (identical) reads present a challenge because they can arise from independent DNA fragments or by PCR amplification of a single fragment. In the former case, the duplicate reads are signals, in the latter case they are noise (experimental artefact). A safe solution is to keep a fixed number of hits per genomic location (considering different strands as different locations) according to sequencing depth, and in this way better specificity (fewer false positive peaks) can be achieved [8]. However in terms of estimating the protein's affinity for a given genomic region, it is more reasonable to consider all hits. This can be done in a well-designed pipeline with certain steps before and after peak calling. For example, one can remove a certain number of duplicates to call confident peaks, and then put duplicates back to refine properties of these peaks such as peak height and boundaries.

### Assessment of Reproducibility

To ensure that experimental results are reproducible, it is recommended to perform at least two biological replicates of each ChIP-seq experiment and examine the reproducibility of both the reads and identified peaks [7], [24]. The reproducibility of the reads can be measured by computing the Pearson correlation coefficient (PCC) of the (mapped) read counts at each genomic position [25]. The range of PCC is typically from 0.3–0.4 (for unrelated samples) to >0.9 (for replicate samples in high-quality experiments). Low values typically suggest one or both replicates may be of low quality. However, this quantity can be dominated by a small number of very highly enriched regions, so it may not reflect the reproducibility for regions that are less enriched [26]. Thus it is important to remove the artefact regions with high ChIP signals, such as regions near centromeres, telomeres, satellite repeats, and ENCODE and 1000 Genomes blacklisted regions, before computing the PCC. To measure the reproducibility at the level of peak calling, IDR analysis (**Box 8**) [23] can be applied to the two sets of peaks identified from a pair of replicates. This analysis assesses the rank consistency of identified peaks between replicates, and outputs the number of peaks that pass a user-specified reproducibility threshold (e.g., IDR = 0.05). It has been reported that using a reproducibility-based metric (e.g., IDR) rather than an enrichment-based metric (e.g., FDR or *p*-value) makes the numbers of peaks declared more comparable across experiments [7]. In addition, IDR analysis can also be used for comparing and selecting peak callers [8], [23] and identifying experiments with low quality [7].

**Box 8. Irreproducible discovery rate (IDR)**

Given a set of peak calls for a pair of replicate data sets, the peaks can be ranked based on a criterion of significance, such as the *p*-value, the *q*-value, or the fold enrichment. Significant peaks generally are ranked more consistently across the replicates than the peaks with low significance. This provides an indicator of the transition from real signal to noise. IDR [23] quantifies this transition by classifying peaks into a reproducible and an irreproducible group, where the peaks in the reproducible group should be ranked higher and more consistently across replicates than the irreproducible group (**Figure S2**). It assigns each signal a reproducibility index, which estimates its probability to be reproducible, and also reports the expected rate of irreproducible discoveries in the selected peaks (referred to as IDR) in a fashion analogous to that of false discovery rate (FDR). An R package for computing IDR is given in [23]. Prototypical examples illustrated using ENCODE data may be found in [7]. When using IDR, a relatively relaxed peak-calling threshold is advised because the IDR algorithm requires sampling of both signal and noise distributions to assess the reproducibility of peaks.

A major advantage of the IDR method is that it is independent of the peak-calling algorithms and can be applied to a variety of significance criteria, across labs and platforms. It has been shown that it produces a stable threshold that is more consistent across laboratories, antibodies, and analysis protocols (e.g., peak callers) than FDR measures [7].

### Differential Binding Analysis

Comparative ChIP-seq analysis of an increasing number of protein-bound regions across conditions or tissues is expected with the steady raise of NGS projects. For example, temporal or developmental designs of ChIP-seq experiments can provide different snapshots of a binding signal for the same TF, uncovering stage-specific patterns of gene regulation [27], [28]. With this in mind, one should note that the simple binary overlap of two sets of peaks (e.g., BEDTools [29]) does not represent the optimal approach when comparing peaks [25].

Two alternatives have been proposed. The first one—qualitative—implements hypothesis testing on multiple overlapping sets of peaks [30], therefore extending the two-set overlap approach mentioned above. The second one—quantitative—proposes the analysis of differential binding between conditions based on the total counts of reads in peak regions or on the read densities, i.e., counts of reads overlapping at individual genomic positions (**Table S3** and [31], [32]). The direct calculation of differentially bound regions between treatment samples without controls (i.e., using one of them as a control) is not recommended because highly enriched regions could be identified due to artefacts or different chromatin structure and not due to true binding events.

Typically, both methodologies assume that significant (see section “Peak Calling”) and reproducible (see section “Assessment of Reproducibility”) peaks have been found in advance independently for each condition. In order to increase sensitivity for detecting differentially bound regions (at the expense of increasing the number of false positives), more relaxed thresholds can be used to find peaks at each condition. Then, depending on the biological question, the sets of peaks called in any of the conditions can be considered separately, or collapsed into one or more meaningful lists of consensus peak regions. One can use the qualitative approach to get an initial overview of differential binding. However, peaks identified in all conditions will never be declared as differentially bound sites by this approach based just on the positions of the peaks [33]. The quantitative approach works with read counts (e.g., DBChIP [33]) or read densities (e.g., MAnorm [34]) computed over peak regions, and has higher computational cost, but is recommended as it provides precise statistical assessment of differential binding across conditions (e.g., *p*-values or *q*-values linked to read-enrichment fold changes). It is strongly advised to verify that the data fulfill the requirements of the software chosen for the analysis. For instance, DIME [35] assumes that a significant proportion of peaks are common to the conditions under comparison, MAnorm assumes that peaks that are common in both conditions do not change significantly, while other methodologies may expect a constant number of peaks across conditions [25]. Importantly, with some tools only two conditions can be submitted simultaneously for comparison (e.g., MAnorm), and some may perform better depending on the protein ChIPed (e.g., ChIPDiff [36] for histone marks and POLYPHEMUS [37] for RNA Pol II).

### Peak Annotation

The aim of the annotation is to associate the ChIP-seq peaks with functionally relevant genomic regions, such as gene promoters, transcription start sites, intergenic regions, etc. In the first step, one uploads the peaks and reads (in an appropriate format, e.g., BED or GFF for peaks, WIG or bedGraph for normalized read coverage; see **Text S1** and [38]–[41]) to a genome browser, where regions can be manually examined in search for associations with annotated genomic features. If comparable data (e.g., ChIP-qPCR) is available, it can be compared with the ChIP-seq peaks and reads manually in the browser as well. A systematic analysis can also be performed using tools in packages such as BEDTools to compute the distance from each peak to the nearest landmark (e.g., TSS), or to identify the genes within a given distance of a peak. The output of such “location analyses,” obtained for instance using CEAS [42] or the Bioconductor package ChIPpeakAnno [43], can be further correlated with expression data (e.g., to determine if proximity of a gene to a peak is correlated with its expression) or subjected to a gene ontology analysis (e.g., to determine if the ChIPed protein is involved in particular biological processes). Gene ontology analysis can be done using DAVID [44], GREAT [45], or GSEA [46]. Sometimes, the reads densities relative to a specific annotated feature are plotted and compared across different samples, thus revealing protein-binding pattern differences between them [47].

### Motif Analysis

Motif analysis is useful for much more than just identifying the causal DNA-binding motif in TF ChIP-seq peaks. When the motif of the ChIPed protein is already known, motif analysis provides validation of the success of the experiment. Even when the motif is not known beforehand, identifying a centrally located motif in a large fraction of the peaks by motif analysis is indicative of a successful experiment. Motif analysis can also identify the DNA-binding motifs of other proteins that bind in complex or in conjunction with the ChIPed protein, illuminating the mechanisms of transcriptional regulation. Motif analysis is also useful with histone modification ChIP-seq because it can discover unanticipated sequence signals associated with such marks. **Table S4** and [48], [49] list a small sample of the publicly available tools for motif analysis.

Motif analysis is applied to the genomic regions identified by peak-calling algorithms. Hence, the first step in motif analysis is to assemble a set of genomic sequences in FASTA format corresponding to all the significant ChIP-seq peaks [50]–[54]. The second step in motif analysis is motif discovery and it is advisable to input the peak sequences to two or more of the many algorithms able to discover sequence motifs in unaligned DNA sequences [55]–[58], as the algorithms have complementary strengths and weaknesses. Some motif discovery algorithms form part of pipelines that perform several motif analysis steps (e.g., MEME-ChIP [57] and peak-motifs [58]), including word-based motif discovery algorithms and motif enrichment algorithms that can identify motifs present in only a small fraction of the peaks. Following motif discovery, comparing the discovered motifs with known DNA motifs using motif comparison software [59], [60] is useful to confirm the presence of the ChIPed TF motif if its (or its TF-family) binding motif is known. The results will also provide hints about other TFs that bind near the ChIPed TF. Next, central motif enrichment analysis will determine if other known DNA motifs are enriched near the centers (or summits) of the ChIP-seq peaks [61]. It can also be useful to perform local motif enrichment analysis on regions centered on genomic landmarks such as transcription start sites overlapped by ChIP-seq peaks [61]. Additionally, a motif spacing analysis detects preferred distances and arrangements of pairs of motifs that can be indicative of physical interactions between TFs [62]. Finally, motif prediction maps and visualizes the genomic locations of the motifs in each of the ChIP-seq regions [63], [64]. In this step, the discovered or enriched motifs are used to scan the ChIP-seq peak regions, and the coordinates of the matches are uploaded to a genome browser for visualization.

## Outlook

The challenges of ChIP-seq require novel experimental, statistical, and computational solutions. Ongoing advances will allow ChIP-seq to analyze samples containing far fewer cells, greatly expanding its applicability in areas such as embryology and development where large samples are prohibitively expensive or difficult to obtain. Nano-ChIP-seq can analyze a sample as small as 10,000 cells [65]. No less critical is to trim today's peaks that are much wider than the actual transcription factor binding sites. This is necessary to distinguish artefacts from bona fide joint binding events: most transcription factors competitively, cooperatively, or co-bind with other transcription factors, the transcriptional machinery, or cofactors. The effects of context-dependent regulatory mechanisms can fundamentally differ from the effects of individual binding events [66]. To address this issue, the Genome Positioning System (GPS) resolves closely spaced peaks using a segmented expectation maximization algorithm [67]. A promising experimental method for localizing narrow peaks is ChIP-exo that uses bacteriophage λ exonuclease to digest the ends of DNA fragments not bound to protein [68].

The number of false positive peaks can be reduced both experimentally and computationally. Improving antibody specificity is a long-term endeavor, and despite impressive progress, still a quarter of histone modification antibodies fail the specificity test [69]. Another way to eliminate massive amounts of false positive peaks is to limit the regulatory binding sites to nucleosome-depleted regions, which are accessible for regulator binding. These regions are mapped by DNase I hypersensitivity sequencing (DNase-seq) and similar techniques: Thurman et al. found that 94% of the human transcription factor binding sites fell into DNase hypersensitivity regions with only a few exceptions like the transcription factors ZNF274, KAP1, and SETDB1, which also bind to closed chromatin [70]. False positive peaks are also due to unrealistic *p*-values (and hence FDRs) coming from unrealistic statistical models used in most methods [71]. The computational analysis of peak calling is still in its infancy, expanding the diverse and condition-specific performance of the methods [72], [73], therefore we recommend using several methods for peak calling.

Perhaps the most important novel developments are related to the detection and analyses of distal regulatory regions, which are distant in sequence but brought close in 3-D space by DNA bending. To reveal such 3-D mechanisms of transcriptional regulation, two major techniques have emerged: chromatin interaction analysis by paired-end tags (CHIA-PET) [74] and chromosome conformation capture assays such as circular chromosome conformation capture (4C) [75] or chromosome conformation capture carbon copy (5C) [76].

Biological functions of binding sites are not necessarily indicated by the reproducibility of peaks or FDR/IDR values (**Box 8**, [7], [23], [77], [78]). This issue re-emerged during the ENCODE Project that produced unprecedented regulatory information [66], [79] under rigorous quality standards [7]. DNA-protein binding is dynamic, and the measured strength of a binding event depends (among other things) on the fraction of cells in the (often inhomogeneous) sample where it occurs, as well as the proportion of the time it is occupied in a given cell. Hence, “weak” binding sites, regardless of what significance threshold is used, may have strong biological functions [80]–[82]. ChIP-seq will also detect *indirect* DNA binding by the protein (via another protein or complex), so predicted sites *not* containing the motif may also be functional. Finally, binding does not necessarily imply function, so it will remain necessary to use additional information (such as expression or chromatin conformation data) to reliably infer the function of individual binding events [83].

The diverse experimental and computational methods discussed here are revolutionizing our understanding of the complex networks that, by regulating transcription, impact translation and almost all biological processes.

## Supporting Information

Text S1 **Standard graphing track data formats for genome browser visualization.**
(DOCX)

Figure S1 **Assessment of read quality using strand cross-correlation.** Strand cross-correlation is computed as the Pearson correlation between the positive and the negative strand profiles at different strand shift distances, *k*. The cross-correlation (panel A) usually peaks at two distances of shift, one corresponding to the read length, and one to the average fragment length of the library. The absolute and relative height of the two peaks is useful for assessing IP enrichment. Adapted from Landt et al. [7].
(TIF)

Figure S2 **The irreproducible discovery rate (IDR) framework for assessing reproducibility of ChIP-seq data sets.** Panel A shows a scatterplot of the significance scores of peaks identified in two replicate ChIP-seq experiments. The IDR method classifies peaks into reproducible (black) and irreproducible (red) groups, and computes for each peak the probability that the peak belongs to the irreproducible group. It ranks and selects peaks according to this probability, and computes IDR, the expected rate of irreproducible discoveries in the selected peaks. Panel B shows the estimated IDR at different rank thresholds when the peaks are sorted by the original significance score.
(TIF)

Table S1 **Examples of peak callers employed in ChIP-seq.** The list includes tools that allow the processing and post-processing of diverse types of narrow read-enriched regions (peaks), broad enriched regions (domains), and mixed signals such as in RNA Pol II ChIP-seq.
(DOCX)

Table S2 **Normalization methods for the comparative analysis of ChIP-seq data sets.**
(DOCX)

Table S3 **Software packages for the analysis of differential binding in ChIP-seq.** The table shows examples of algorithms available for differential binding analysis using ChIP-seq data.
(DOCX)

Table S4 **Software tools for motif analysis of ChIP-seq peaks and their uses.** The table gives examples of publicly available software tools for performing motif analysis on ChIP-seq peaks or nearby genes. The tools are grouped by the major task (“category”), and checkmarks indicate the specific steps that each tool performs. Web-based motif discovery input size limits—ChIPMunk: unknown; CompleteMOTIFS: 500,000 base pairs; MEME-ChIP: 50,000,000 base pairs; peak-motifs: no limit; Cistrome: 5,000 peaks.
(DOCX)
