# Supplementary material for: Practical Guidelines for the Comprehensive Analysis of ChIP-seq Data
Source: PLoS Comput Biol. 2013 Nov 14;9(11):e1003326. doi: 10.1371/journal.pcbi.1003326 (PMC3828144; doi:10.1371/journal.pcbi.1003326)
Supplement: Text S1 — Standard graphing track data formats for genome browser visualization. (DOCX) [file pcbi.1003326.s001.docx]

**Text S1. Standard graphing track data formats for genome browser visualization.**

Aligned reads in standard formats (e.g., SAM/BAM) can be visualized in a genome browser [38-40], together with the reference annotation of the organism (if available), and Browser Extensible Data (BED) files with ChIP-seq peak locations. However, Wiggle (WIG) or bedGraph track data formats enable a more appropriate drawing of the read coverage on the genome browser (frequently the length of each read is extended to the average fragment size of the experiment in the 3' direction). Taking into account the structure of the data in selecting the visualization format is essential to avoid unnecessary large data files, allowing an efficient and quick display. When the data is spaced at regular intervals WIGs are preferred, with fixed step WIGs being more efficient than variable step ones. If the regions do not have constant size and are spaced at irregular intervals bedGraph is recommended. Compressed binary indexed versions of WIG and bedGraph formats (BigWig), and of BED (BigBed) store the data more efficiently than their equivalents, with the advantage that only the data in the current view is transmitted for visualization [41]. A description of the data file formats accepted by the UCSC Genome Browser, as well as utilities for creation and format conversion can be found at http://genome.ucsc.edu/FAQ/FAQformat.html.

Many peak callers employed in ChIP-seq (Section 3) are able to produce BED files with the localization of peaks, together with WIG or bedGraph track data files to represent the number of tags that overlap at each nucleotide position (pileup aligned reads with a user-settable extension size), and/or genome-wide scores produced by the statistical model used. For example, PeakRanger [93] is able to generate compressed and uncompressed bedGraph or variable-step WIG files of unnormalized extended read pileup. MACS [17] (version 2.0.10) can generate four types of bedGraph files storing: the fragment pileup, local lambda values from control sample, Poisson *p*-value scores, and *q*-value scores from Benjamini–Hochberg–Yekutieli procedure. CSAR [85] produces a single WIG file of read-enrichment scores based on the Poisson distribution or ratio between a treatment sample and its paired control. Importantly, normalized coverage tracks are essential to compare several samples in the genome browser. It can be done by normalizing the read pileup by the total number of mapped reads in the library per, e.g., 1 million reads [25].
