# Supplementary figures and images for: Practical Guidelines for the Comprehensive Analysis of ChIP-seq Data
Source: PLoS Comput Biol. 2013 Nov 14;9(11):e1003326. doi: 10.1371/journal.pcbi.1003326 (PMC3828144; doi:10.1371/journal.pcbi.1003326)

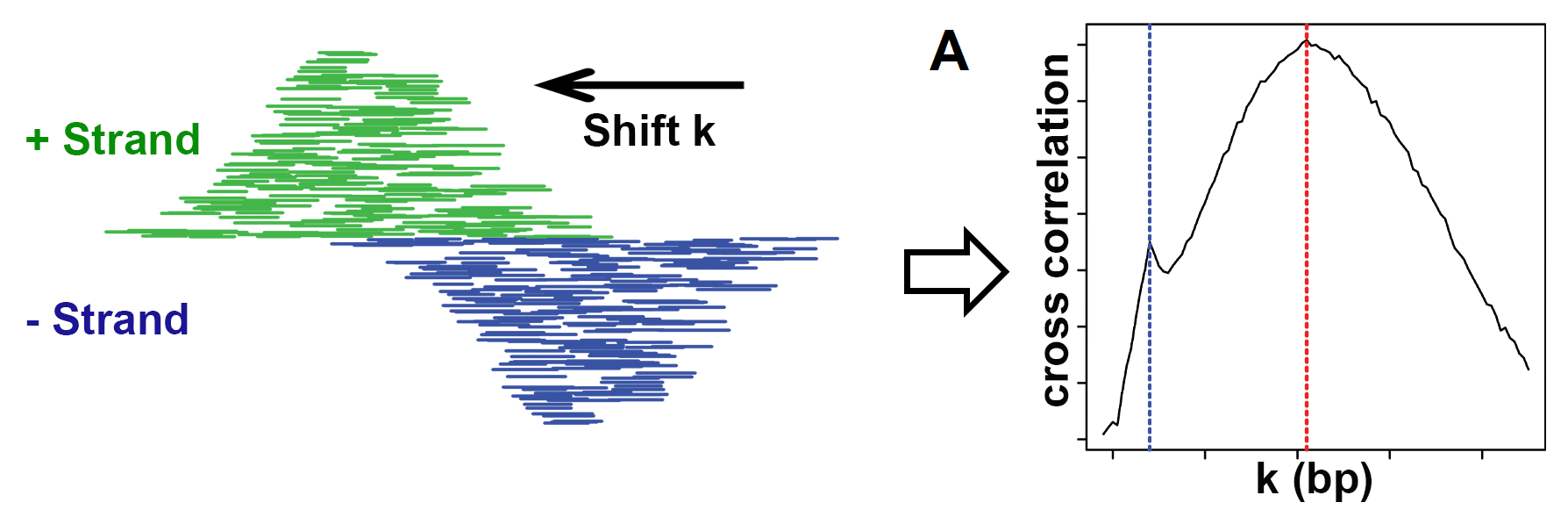

Supplement: Figure S1 — Assessment of read quality using strand cross-correlation. Strand cross-correlation is computed as the Pearson correlation between the positive and the negative strand profiles at different strand shift distances, k. The cross-correlation (panel A) usually peaks at two distances of shift, one corresponding to the read length, and one to the average fragment length of the library. The absolute and relative height of the two peaks is useful for assessing IP enrichment. Adapted from Landt et al. [7]. (TIF) [file pcbi.1003326.s002.tif]

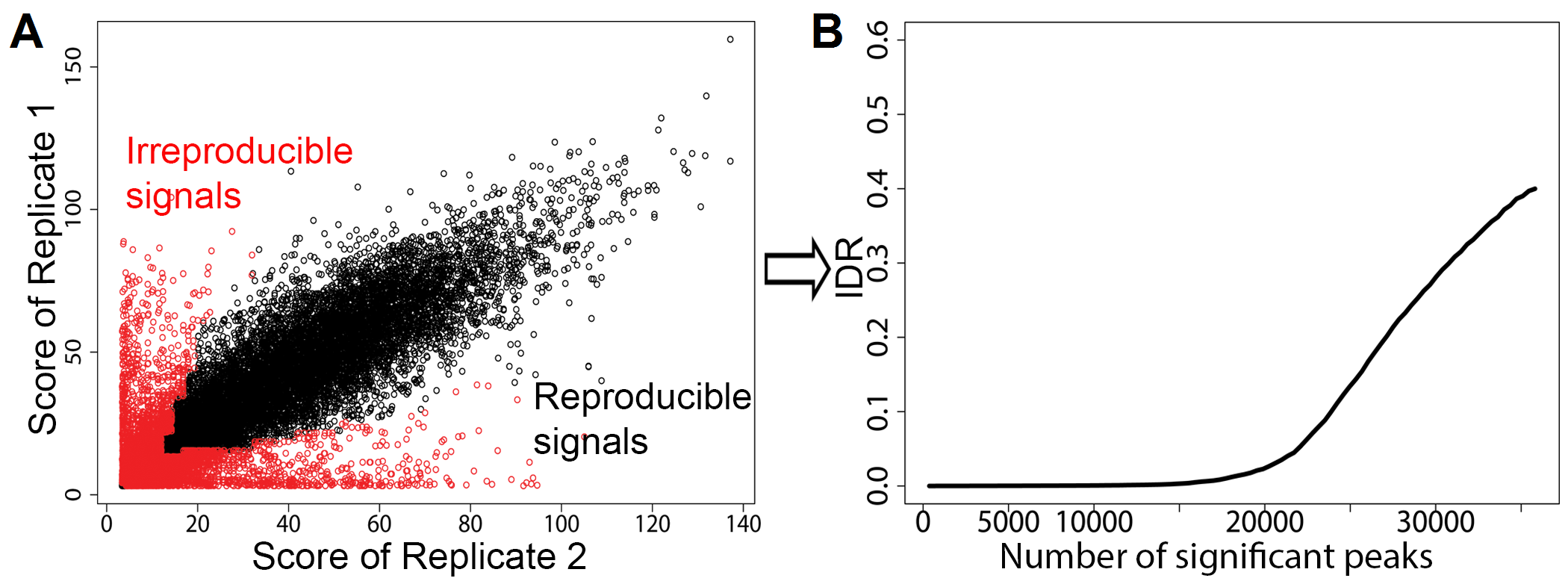

Supplement: Figure S2 — The irreproducible discovery rate (IDR) framework for assessing reproducibility of ChIP-seq data sets. Panel A shows a scatterplot of the significance scores of peaks identified in two replicate ChIP-seq experiments. The IDR method classifies peaks into reproducible (black) and irreproducible (red) groups, and computes for each peak the probability that the peak belongs to the irreproducible group. It ranks and selects peaks according to this probability, and computes IDR, the expected rate of irreproducible discoveries in the selected peaks. Panel B shows the estimated IDR at different rank thresholds when the peaks are sorted by the original significance score. (TIF) [file pcbi.1003326.s003.tif]
