# Supplementary material for: Practical Guidelines for the Comprehensive Analysis of ChIP-seq Data
Source: PLoS Comput Biol. 2013 Nov 14;9(11):e1003326. doi: 10.1371/journal.pcbi.1003326 (PMC3828144; doi:10.1371/journal.pcbi.1003326)
Supplement: Table S1 — Examples of peak callers employed in ChIP-seq. The list includes tools that allow the processing and post-processing of diverse types of narrow read-enriched regions (peaks), broad enriched regions (domains), and mixed signals such as in RNA Pol II ChIP-seq. (DOCX) [file pcbi.1003326.s004.docx]

**Table S1. Examples of peak callers employed in ChIP-seq.**

| **Software tool** | **Version** | **Availability** | **Point- source (peaks)** | **Broad regions (domains)** |
| --- | --- | --- | --- | --- |
| **BayesPeak [88]** | 1.10.0 | http://bioconductor.org/packages/release/bioc/html/BayesPeak.html | Yes |  |
| **BEADS**^§^ **[84]** | 1.1 | http://beads.sourceforge.net/ | Yes | Yes |
| **CCAT [91]** | 3.0 | http://cmb.gis.a-star.edu.sg/ChIPSeq/paperCCAT.htm |  | Yes |
| **CisGenome [56]** | 2.0 | http://www.biostat.jhsph.edu/~hji/cisgenome/ | Yes |  |
| **CSAR [85]** | 1.10.0 | http://bioconductor.org/packages/release/bioc/html/CSAR.html | Yes |  |
| **dPeak** | 0.9.9 | http://www.stat.wisc.edu/~chungdon/dpeak/ | Yes |  |
| **GPS/GEM [67,18]** | 1.3 | http://cgs.csail.mit.edu/gps/ | Yes |  |
| **HPeak [87]** | 2.1 | http://www.sph.umich.edu/csg/qin/HPeak/ | Yes |  |
| **MACS [17]** | 2.0.10 | https://github.com/taoliu/MACS/ | Yes | Yes |
| **NarrowPeaks**^§^ | 1.4.0 | http://bioconductor.org/packages/release/bioc/html/NarrowPeaks.html | Yes |  |
| **PeakAnalyzer/** **PeakSplitter**^§^ **[89]** | 1.4 | http://www.bioinformatics.org/peakanalyzer | Yes |  |
| **PeakRanger [93]** | 1.16 | http://ranger.sourceforge.net/ | Yes | Yes |
| **PeakSeq [24]** | 1.1 | http://info.gersteinlab.org/PeakSeq | Yes |  |
| **polyaPeak**^§^ | 0.1 | http://web1.sph.emory.edu/users/hwu30/polyaPeak.html | Yes |  |
| **RSEG [92]** | 0.6 | http://smithlab.usc.edu/histone/rseg/ |  | Yes |
| **SICER [90]** | 1.1 | http://home.gwu.edu/~wpeng/Software.htm |  | Yes |
| **SIPeS [21]** | 2.0 | http://gmdd.shgmo.org/Computational-Biology/ChIP-Seq/download/SIPeS | Yes |  |
| **SISSRs [19]** | 1.4 | http://sissrs.rajajothi.com/ | Yes |  |
| **SPP [9]** | 1.1 | http://compbio.med.harvard.edu/Supplements/ChIP-seq/ | Yes | Yes |
| **USeq [97]** | 8.5.1 | http://sourceforge.net/projects/useq/ | Yes |  |
| **ZINBA [86]** | 2.02.03 | http://code.google.com/p/zinba/ | Yes | Yes |

^§^ Only for post-processing.
