# Supplementary material for: Practical Guidelines for the Comprehensive Analysis of ChIP-seq Data
Source: PLoS Comput Biol. 2013 Nov 14;9(11):e1003326. doi: 10.1371/journal.pcbi.1003326 (PMC3828144; doi:10.1371/journal.pcbi.1003326)
Supplement: Table S2 — Normalization methods for the comparative analysis of ChIP-seq data sets. (DOCX) [file pcbi.1003326.s005.docx]

**Table S2. Normalization methods for the comparative analysis of ChIP-seq data sets.**

| **Linear Algorithms** | Focus on negative control sample | Publication Time |
| --- | --- | --- |
| **PeakSeq [24]** | Yes | Jan 2009 |
| **Cisgenome [56]** | Yes | Nov 2008 |
| **MACS [17]** | Yes | Sept 2008 |
| **USeq [97]** | Yes | Dec 2008 |
| **RPKM [98]** | No | May 2008 |

| **Non-linear Algorithms** | Focus on negative control sample | Publication Time |
| --- | --- | --- |
| **Locally weighted regression with respect to mean and variance [28]** | No | Jan 2009 |
| **MAnorm [34]** | No | March 2012 |
| **POLYPHEMUS [37] (Quantile and locally weighted regression)** | No | Feb 2012 |
