# Supplementary material for: Practical Guidelines for the Comprehensive Analysis of ChIP-seq Data
Source: PLoS Comput Biol. 2013 Nov 14;9(11):e1003326. doi: 10.1371/journal.pcbi.1003326 (PMC3828144; doi:10.1371/journal.pcbi.1003326)
Supplement: Table S3 — Software packages for the analysis of differential binding in ChIP-seq. The table shows examples of algorithms available for differential binding analysis using ChIP-seq data. (DOCX) [file pcbi.1003326.s006.docx]

**Table S3. Software packages for the analysis of differential binding in ChIP-seq**.

| **Software tool** | **Availability** | **Notes** |
| --- | --- | --- |
| **ChIPDiff [36]** | http://cmb.gis.a-star.edu.sg/ChIPSeq/paperChIPDiff.htm | Differential histone modification sites using a hidden Markov model |
| **Comparative ChIP-seq [25]** | http://www.starklab.org/data/bardet_natprotoc_2011/ | Fold change ratio between normalized peak heights |
| **DBChIP [33]** | http://pages.cs.wisc.edu/~kliang/DBChIP/ | Assigns uncertainty measures in a test of non-differential binding (uses edgeR) |
| **DESeq**^§^ **[31]** | http://www.bioconductor.org/packages/release/bioc/html/DESeq.html | Test based on a model using the negative binomial distribution |
| **DiffBind** | http://www.bioconductor.org/packages/release/bioc/html/DiffBind.html | Differential binding affinity analysis (uses edgeR and DESeq) |
| **DIME [35]** | http://cran.r-project.org/web/packages/DIME/ | Differential identiﬁcation using mixtures ensemble |
| **edgeR**^§^ **[32]** | http://www.bioconductor.org/packages/release/bioc/html/edgeR.html | Empirical Bayes estimation and exact tests based on the negative binomial distribution |
| **MACS [17] (version 2)** | https://github.com/taoliu/MACS/ | Differential peak detection based on paired four bedGraph files |
| **MAnorm [34]** | http://bcb.dfci.harvard.edu/~gcyuan/MAnorm/MAnorm.htm | Robust regression to derive a linear model |
| **MMDiff** | http://bioconductor.org/packages/release/bioc/html/MMDiff.html | Differences in shape using Kernel methods |
| **NarrowPeaks** | http://bioconductor.org/packages/release/bioc/html/NarrowPeaks.html | Shape-based analysis of variation using functional PCA |
| **POLYPHEMUS [37]** | http://cran.r-project.org/web/packages/polyphemus/ | Non-linear normalization on RNA Pol II profiling |

^§^ Originally developed for gene expression count data.
