# Supplementary material for: Practical Guidelines for the Comprehensive Analysis of ChIP-seq Data
Source: PLoS Comput Biol. 2013 Nov 14;9(11):e1003326. doi: 10.1371/journal.pcbi.1003326 (PMC3828144; doi:10.1371/journal.pcbi.1003326)
Supplement: Table S4 — Software tools for motif analysis of ChIP-seq peaks and their uses. The table gives examples of publicly available software tools for performing motif analysis on ChIP-seq peaks or nearby genes. The tools are grouped by the major task (“category”), and checkmarks indicate the specific steps that each tool performs. Web-based motif discovery input size limits—ChIPMunk: unknown; CompleteMOTIFS: 500,000 base pairs; MEME-ChIP: 50,000,000 base pairs; peak-motifs: no limit; Cistrome: 5,000 peaks. (DOCX) [file pcbi.1003326.s007.docx]

**Table S4. Software tools for motif analysis of ChIP-seq peaks and their uses.**

| ***Category*** | ***Software tool*** | ***Web Server*** | ***Obtain peak regions*** | ***Motif discovery*** | ***Motif comparison*** | ***Central motif enrichment analysis*** | ***Local motif enrichment analysis*** | ***Motif spacing analysis*** | ***Motif prediction/mapping*** |
| --- | --- | --- | --- | --- | --- | --- | --- | --- | --- |
| **Obtaining sequences** | **Galaxy [50-52]** | X | X |  |  |  |  |  |  |
|  | **RSAT [53]** | X | X |  |  |  |  |  |  |
|  | **UCSC Genome Browser [54]** | X | X |  |  |  |  |  |  |
| **Motif discovery + more** | **ChIPMunk [55]** | X |  | X |  |  |  |  |  |
|  | **CisGenome [56]** |  |  | X | X |  |  |  |  |
|  | **CompleteMOTIFS [48]** | X |  | X | X |  |  |  |  |
|  | **MEME-ChIP [57]** | X |  | X | X | X |  |  |  |
|  | **peak-motifs [58]** | X |  | X | X |  |  |  | X |
|  | **Cistrome [49]** | X | X | X |  | X | X |  | X |
| **Motif comparison** | **STAMP [59]** | X |  |  | X |  |  |  |  |
|  | **TOMTOM [60]** | X |  |  | X |  |  |  |  |
| **Motif enrichment/spacing** | **CentriMo [61]** | X |  |  |  | X | X |  |  |
|  | **SpaMo [62]** | X |  |  |  |  |  | X |  |
| **Motif prediction/mapping** | **FIMO [63]** | X |  |  |  |  |  |  | X |
|  | **PATSER [64]** | X |  |  |  |  |  |  | X |
